# An immunoinformatics and structural vaccinology approach to design a novel and potent multi-epitope base vaccine targeting *Zika virus*

**DOI:** 10.1186/s13065-024-01132-3

**Published:** 2024-02-13

**Authors:** Mohammed Ageeli Hakami

**Affiliations:** Department of Clinical Laboratory Sciences, College of Applied Medical Sciences, Shaqra University, Al-Quwayiyah, Riyadh, Saudi Arabia

**Keywords:** Vaccine, Infectious disease, B-cell epitopes, T-cell epitopes, Biomarkers, Toll-like receptor

## Abstract

*Zika virus* is an infectious virus, that belongs to *Flaviviridae* family, which is transferred to humans through mosquito vectors and severely threatens human health; but, apart from available resources, no effective and secure vaccine is present against *Zika virus,* to prevent such infections. In current study, we employed structural vaccinology approach to design an epitope-based vaccine against *Zika virus,* which is biocompatible, and secure and might trigger an adaptive and innate immune response by using computational approaches. We first retrieved the protein sequence from National Center for Biotechnology Information (NCBI) database and carried out for BLAST P. After BLAST P, predicted protein sequences were shortlisted and checked for allergic features and antigenic properties. Final sequence of *Zika virus*, with accession number (APO40588.1) was selected based on high antigenic score and non-allergenicity. Final protein sequence used various computational approaches including antigenicity testing, toxicity evaluation, allergenicity, and conservancy assessment to identify superior B-cell and T-cell epitopes. Two B-cell epitopes, five MHC-six MHC-II epitopes and I were used to construct an immunogenic multi-epitope-based vaccine by using suitable linkers. A 50S ribosomal protein was added at N terminal to improve the immunogenicity of vaccine. In molecular docking, strong interactions were presented between constructed vaccine and Toll-like receptor 9 (− 1100.6 kcal/mol), suggesting their possible relevance in the immunological response to vaccine. The molecular dynamics simulations ensure the dynamic and structural stability of constructed vaccine. The results of C-immune simulation revealed that constructed vaccine activate B and T lymphocytes which induce high level of antibodies and cytokines to combat *Zika* infection. The constructed vaccine is an effective biomarker with non-sensitization, nontoxicity; nonallergic, good immunogenicity, and antigenicity, however, experimental assays are required to verify the results of present study.

## Introduction

*Zika virus* was first discovered in 1947 in a rhesus monkey in the Uganda forest called the Zika forest [1]. The genome size of the *Zika virus* is 10.7 kb and *Zika virus* is an RNA-based virus. The viral genome contains both structural and non-structural proteins. The envelope-E, Capsid-C, and the pre-membrane /prM are the main structural proteins while the NS1-NS5 is the non-structure protein that is vital for the replication of the virus [2]. In 1953, the *Zika virus* in humans was reported in Nigeria and the virus was confirmed in three individuals [3]. After that, some other human cases were reported in Asia and Africa after the virus' initial screening. Numerous non-human primates have developed antibodies against *Zika virus*, but in the absence of other primates, humans are primarily considered to be its host [4]. The *Zika virus*, like other flaviviruses, spread by many mosquito species and belongs to the family of Flaviviridae viruses. In over 80% of cases, *Zika virus* infections are asymptomatic, and the majority of patients only exhibit minor symptoms [5]. *Zika virus* is most frequently spread by a mosquito bite, where the initial infection most likely takes place in human skin cells and affects immature dendritic cells, permissive human dermal fibroblasts, and epidermal keratinocytes [6]. Direct sexual contact is considered another important human *Zika virus* transmission route in addition to mosquito bites[7]. Microcephaly in newborn infants is caused by *Zika virus* [8].Headache, extreme tiredness, malaise, arthralgia, conjunctivitis, and fever are among the *Zika virus* symptoms [9]. In the past, the clinical state caused by *Zika virus* in humans was described as a mild influenza-like sickness that recovered within days and affected 20% of the individuals [10]. However, French Polynesia had a greater proportion of symptomatic infections (about 50%). Fever, arthralgia, myalgia, conjunctivitis, headache, and rash are the most common symptoms of the *Zika virus* infections that occurs within 3–7 days following a mosquito bite [11]. *Zika virus* infection has also been linked to serious illnesses in adults, such as multi-organ failure [12], meningitis and encephalitis [13], and thrombocytopenia [14]. *Zika virus* normally does not cause fatal disease in adults, however, it was reported that the *Zika virus* causes death in some cases such as children with sickle cell disease, adults with cancer, and Guillain-Barré syndrome [15]. The development of a vaccine has been facilitated by the close resemblance of *Zika virus* to other well-studied Flaviviruses like the West Nile Virus, Promote Encephalitis Virus, Dengue virus, and tick-borne encephalitis virus [16]. A previous study stated that antibodies that bind to envelope protein may be used to treat *Zika virus* [17]. Here are currently several vaccine candidates under development. Effective vaccinations and targeted medications to treat *Zika virus* infection are still lacking. However, several preventative measures have been suggested to combat *Zika virus* infections, including drinking enough water to prevent dehydration and using paracetamol to reduce pain and fever. These precautions are not sufficient to prevent *Zika virus* transmission, so there is a serious need to search for effective drugs and to develop effective vaccines [18]. Compared to the experimental procedure computational approaches are inexpensive and less time taking [19]. Various bioinformatics tools have been used by researchers for the development of vaccines against *H. pylori* [20], and monkeypox *virus* [21], The aim of current study is to combine multiple B -cell and T-cell epitopes to create a vaccine against the *Zika virus*. The structure of the developed vaccine was modelled and validated using different online tools. Further, molecular docking analysis and molecular dynamic simulation were also carried out to examine the stability of the vaccine construct and its interactions with the host cell receptor. This research will serve as a way to creating a commercial vaccine to combat the present *Zika virus*-associated infections.

## Methodology

### Sequence retrieval

A protein sequence of *Zika virus* was retrieved from NCBI database (https://www.ncbi.nlm.nih.gov/genbank/) [22], with the accession number (6DFI_E) and carried out for protein–protein blast, resulting in top six sequences along with reference sequence as a FASTA format. Furthermore, multiple sequence alignment was performed with the help of MUSCLE v3.6 program [23], and Phylogenetic analysis was carried out through an online server, Mega X. The online tool VexiJen 2.0 [21] (http://www.ddgpharmfac.net/vaxijen/VaxiJen/VaxiJen.html) predicted the antigenicity of each protein. Furthermore, the AllerTOP v.2 server [24]. (https://www.ddg-pharmfac.net/AllerTOP/) (http;//web.expsy. Org.protparam/) was used to estimate the allergenicity of proteins that have been designated as viral antigens. For the subsequent design of the vaccine, antigenic proteins that were also anticipated as nonallergic were processed.

**Fig. 1:**
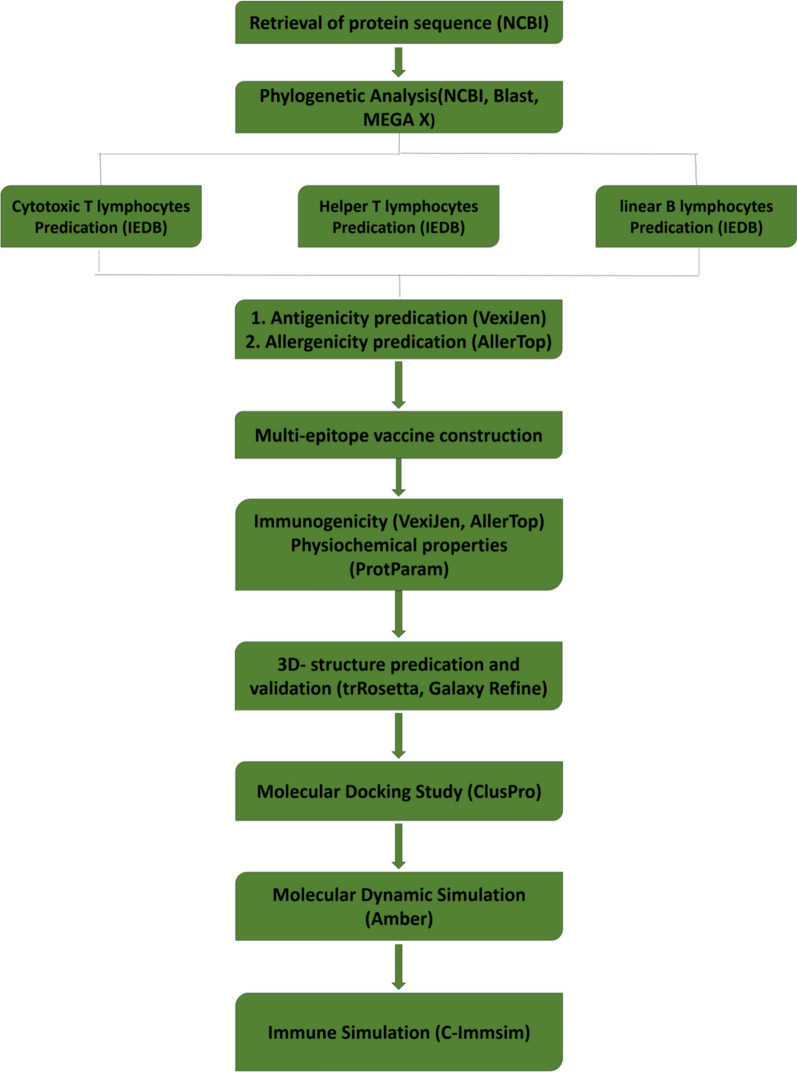
The overall workflow of the constructed vaccine

In addition, the physio-chemical characteristics were predicted using Expasy Protparam (http;//web.expsy. Org.protparam/) [25] and secondary structure was determined by using PSIPRED (http;//bioinf.cs.ucl.ac.uk/psipred) web server [26]. The workflow of the overall work is shown in Fig. 1.

### Prediction of immunogenic B cell and T-cell epitopes

The essential B and T-cell epitopes were then mapped by using the IEDB tool [27], (https://iedb.org/) to predict the B-cell epitopes and T-cell epitopes. The discovery of T cell epitopes were recognized by the MHC-I and MHC-II [28].

A threshold value was set to 0.5, also the IC50 score was assigned through an online server. The < 0.5 value indicates good epitope candidate. However, the binding affinity and IC50 score were inversely proportional to each other, (a small IC50 value presents strong binding affinity towards MHC-MHC-II and I).

### Designing of multi-epitopes vaccine (MVC)

Few other elements such as an adjuvant and appropriate linkers, are required for a successful vaccine construct along with the B and T cell epitopes [29]. There were GPGPG, AAY and EAAAK linkers, which were utilized for vaccine construction. These linkers reduce the vaccine’s immunogenicity, prevent the epitopes from folding on themselves, and enhance the attachment of one epitope to another. Together, these components develop the vaccination sequence for *Zika virus*. The constructed vaccine is comprised of B-cell and T-cell epitopes (MHC-MHC-II epitopes and I) along with linkers and H-tag (given below).
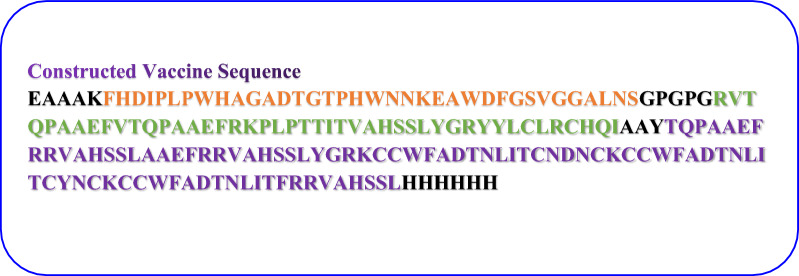


### Antigenicity, allergenicity, physio-chemical properties and secondary structure analysis

The antigenicity and allergenicity of construct vaccine were calculated using VexiJen 2.0 and AllerTOP 2.0, respectively. Thereafter, the molecular weight, Half-life, pI, and other physio chemical properties of the developed vaccine construct were calculated by using Protparam, server [25]. Additionally, the PSIPRED website was employed to determine the secondary structural component [26].

### Modeling the 3D-structure

The three-dimensional structure of the developed vaccine construct was predicted, using the TrRosetta web server [30]. The first model having the lowest estimated error was chosen out of the ten anticipated models that the server generated.

### Validation of the structure

The ERRAT, PRO-CHECK, and ProSA-web servers were used to validate the 3D structure of the developed vaccine. Using the statistics of atom–atom interaction, (non-bonded) ERRAT validates 3D structures. Based on the stereochemical properties of proteins, the PROCHECK tool (http://servicen.mbi.ucla.edu/PROCHECK/) generates a Ramachandran plot for residues [31].

### Molecular docking

The toll-like receptor (TLR) is important for the proper growth of immune response in cells and for the recognition of pathogens [32]. The crystal structure of toll-like receptor 9 (TLR9) was retrieved from RCSB database with PDB; 3WPF. The proposed vaccine was docked to TLR9 to study how immune cell receptors bind to it. The docking was carried out by using the ClusPro server. The tool PDBSum was used to investigate binding interaction [33].

### Molecular dynamic simulation

MD simulation was carried out to explain the stability of vaccine-TLR-9 complex by using Amber software. Different parameters including root mean square deviation (RMSD) and root mean square fluctuation (RMSF) were performed [34].

### C- Immune Simulation

Immune server (http://kraken.iac.rm.cnr.it/C-IMMSIM/index.php) understand the immune simulation of generic of protein sequences in the form of amino acid sequences. This server properly defines the immune system’s cellular and humoral response to vaccine constructs. The C-immune simulation identifies images in which B lymphocytes, cytotoxic T lymphocytes, AB-producer plasma cells, and dendritic cells are presented [35].

## Results

### Protein sequence retrieval for constructed vaccine

**Fig. 2:**
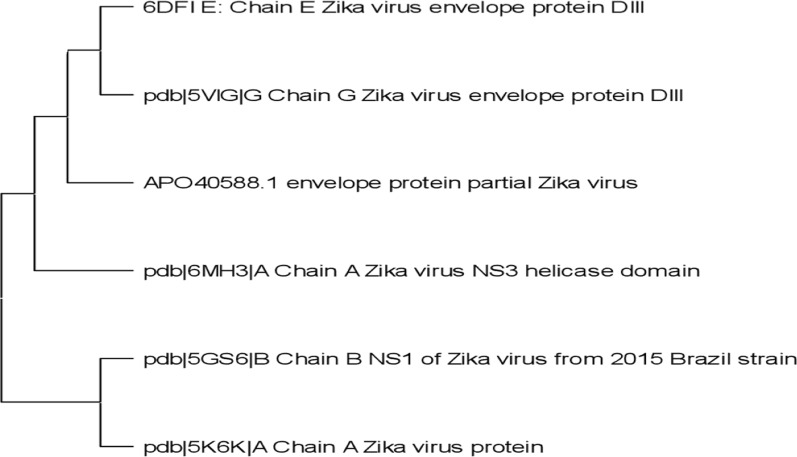
Phylogenetic tree of six protein sequences

The top six protein sequences were selected by performing Protein–Protein Blast (BLAST P), and their allergenicity and antigenicity were checked by using AllerTop and VexiJen 2.0 respectively, as illustrated in **(**Table 1**)**. Then, multiple sequence alignment was conducted with the help of MUSCLE v3.6. A Phylogenetic tree was constructed through MEGA X as shown in **(**Fig. 2**)**. Finally, to construct multi epitope-based vaccine design against *Zika virus,* the protein sequence along with accession ID (APO40588.1) was found to be most antigenic with 0.6205 score and nonallergic.

**Table 1:** The six proteins sequences obtained from NCBI using Protein Blast

Accession Number	Protein Name	Sequence	VexiJen Score	Allergenicity
APO40588.1	Envelope protein, [*Zika virus*]	IRCIGVSNRDFVEGMSGGTWVDVVLEHGGCVTVMAQDKPTVDIELVTTTVSNMAEVRSYCYEASISDMASDSRCPTQGEAYLDKQSDTQYVCKRTLVDRGWGNGCGLFGKGSLVTCAKFACSKKMTGKSIQPENLEYRIMLSVHGSQHSGMIVNDTGHETDENRAKVEITPNSPRAEATLGGFGSLGLDCEPRTGLDFSDLYYLTMNNKHWLVHKEWFHDIPLPWHAGADTGTPHWNNKEALVEFKDAHAKRQTVVVLGSQEGAVHTALAGALEAEMDGAKGRLSSGHLKCRLKMDKLRLKGVSYSLCTAAFTFTKIPAETLHGTVTVEVQYAGTDGPCKVPAQMAVDMQTLTPVGRLITANPVITESTENSKMMLELDPPFGDSYIVIGVGEKKITHHWHRSGSTIGKAFEATVRGAKRMAVLGDTAWDFGSVGGALNSLGKGIHQIFGAAFKSLFGGMSWFSQILIGTLLMWLGLNTKNGSISLMCLALGGVLIFLSTAVSA	0.6205	Non-allergen
DFI_E	Chain E, Zika virus envelope protein DIII	MRLKGVSYSLCTAAFTFTKIPAETLHGTVTVEVQYAGTDGPCKVPAQMAVDMQTLTPVGRLITANPVITESTENSKMMLELDPPFGDSYIVIGVGEKKITHHWHRSGSTI	0.5263	Non-allergen
PDB 5VIG	G Chain G, *Zika virus* envelope protein DIII	MRLKGVSYSLCTAAFTFTKIPAETLHGTVTVEVQYAGTDGPCKVPAQMAVDMQTLTPVGRLITANPVITESTENSKMMLELDPPFGDSYIVIGVGEKKITHHWHRSGSTI	0.5261	Non-allergen
PDB 5GS6	B Chain B, NS1 of *Zika virus* from 2015 Brazil strain	HHHHHHVGCSVDFSKKETRCGTGVFVYNDVEAWRDRYKYHPDSPRRLAAAVKQAWEDGICGISSVSRMENIMWRSVEGELNAILEENGVQLTVVVGSVKNPMWRGPQRLPVPVNELPHGWKAWGKSYFVRAAKTNNSFVVDGDTLKECPLKHRAWNSFLVEDHGFGVFHTSVWLKVREDYSLECDPAVIGTAVKGKEAVHSDLGYWIESEKNDTWRLKRAHLIEMKTCEWPKSHTLWTDGIEESDLIIPKSLAGPLSHHNTREGYRTQMKGPWHSEELEIRFEECPGTKVHVEETCGTRGPSLRSTTASGRVIEEWCCRECTMPPLSFRAKDGCWYGMEIRPRKEPESNLVRSMVTA	0.4533	Non-allergen
> PDB|6MH3|	A Chain A, *Zika virus* NS3 helicase domain	MLKKKQLTVLDLHPGAGKTRRVLPEIVREAIKKRLRTVILAPTRVVAAEMEEALRGLPVRYMTTAVNVTHSGTEIVDLMCHATFTSRLLQPIRVPNYNLNIMDEAHFTDPSSIAARGYISTRVEMGEAAAIFMTATPPGTRDAFPDSNSPIMDTEVEVPERAWSSGFDWVTDHSGKTVWFVPSVRNGNEIAACLTKAGKRVIQLSRKTFETEFQKTKNQEWDFVITTDISEMGANFKADRVIDSRRCLKPVILDGERVILAGPMPVTHASAAQRRGRIGRNPNKPGDEYMYGGGCAETDEGHAHWLEARMLLDNIYLQDGLIASLYRPEADKVAAIEGEFKLRTEQRKTFVELMKRGDLPVWLAYQVASAGITYTDRRWCFDGTTNNTIMEDSVPAEVWTKYGEKRVLKPRWMDARVCSDHAALKSFKEFAAGKR	0.4997	Non-allergen
PDB: 5U04_A	Chain A, Zika virus NS5 RdRp	YHGSYEAPTQGSASSLVNGVVRLLSKPWDVVTGVTGIAMTDTTPYGQQRVFKEKVDTRVPDPQEGTRQVMNIVSSWLWKELGKRKRPRVCTKEEFINKVRSNAALGAIFEEEKEWKTAVEAVNDPRFWALVDREREHHLRGECHSCVYNMMGKREKKQGEFGKAKGSRAIWYMWLGARFLEFEALGFLNEDHWMGRENSGGGVEGLGLQRLGYILEEMNRAPGGKMYADDTAGWDTRISKFDLENEALITNQMEEGHRTLALAVIKYTYQNKVVKVLRPAEGGKTVMDIISRQDQRGSGQVVTYALNTFTNLVVQLIRNMEAEEVLEMQDLWLLRKPEKVTRWLQSNGWDRLKRMAVSGDDCVVKPIDDRFAHALRFLNDMGKVRKDTQEWKPSTGWSNWEEVPFCSHHFNKLYLKDGRSIVVPCRHQDELIGRARVSPGAGWSIRETACLAKSYAQMWQLLYFHRRDLRLMANAICSAVPVDWVPTGRTTWSIHGKGEWMTTEDMLMVWNRVWIEENDHMEDKTPVTKWTDIPYLGKREDLWCGSLIGHRPRTTWAENIKDTVNMVRRIIGDEEKYMDYLSTQVRYGEEGSTPGVL	0.4641	Non-allergen

### Physio chemical characteristics of protein sequence

Human immune systems and functions are significantly affected by the protein physical characteristics [36]. Thereafter, the physical properties of protein were characterized by Expasy ProtParam tool. The protein sequence has 504 amino acids, theoretical PI 6.51, aliphatic index 81.45, and molecular formula C_2395_H_3778_N_660_O_723_S_31_. Furthermore, the estimated Half-life of constructed vaccine comprised of 10 h, 30 min, and 20 h for Escherichia coli, yeast, and mammalian reticulocytes, respectively.

### Assessment of secondary and tertiary structure prediction

**Fig. 3:**
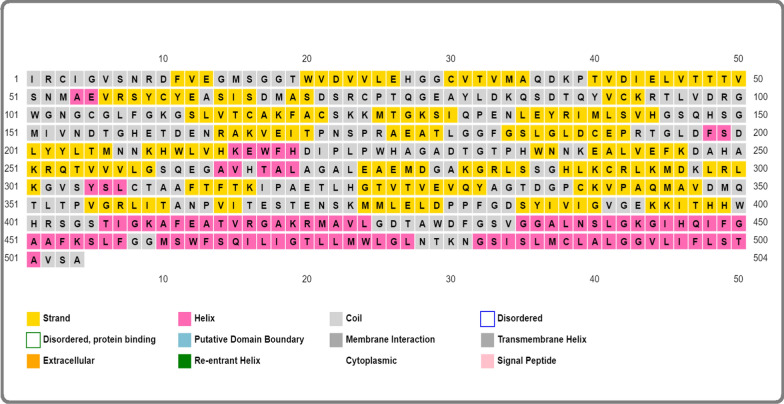
Graphical presentation of protein secondary structure

The secondary structure of *Zika virus,* comprised of beta-sheet (38%), random coil (30%), and alpha helix (32%), predicted by PSIPRED server whereas the three-dimensional structure of protein sequence was determined by trRosetta server **(**Fig. 3**).**

### B cell epitope prediction

**Fig. 4:**
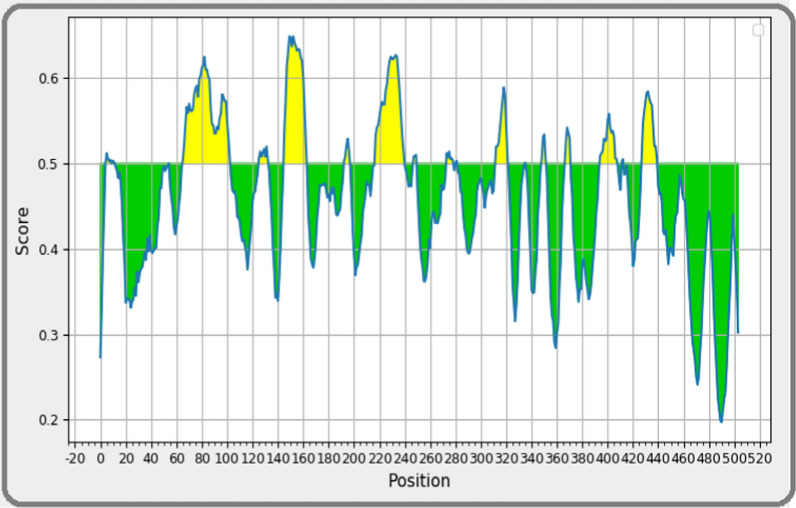
B-Cell Epitopes prediction in selected sequence (yellow color; epitopic area, green color; non-epitopic area)

The selected sequence of *Zika virus* was then used to identify the B cell epitope by using IEDB server. The residues with greater value than threshold (0.5) were selected as B cell epitopes **(**Fig. 4**).** The total seven B cell epitopes were predicted. Finally, based on amino acids sequence, position, length, nonallergic and antigenic score, the top two were selected **(**Table 2**).**

**Table 2:** Bepipred linear epitopes predicted by using IEDB server

Number	Start	End	Peptide	Length
1	218	240	FHDIPLPWHAGADTGTPHWNNKE	23
2	428	440	AWDFGSVGGALNS	13

### T cell epitope

For predicting the MHC-MHC-II, and I the epitopes with IC_50_ less than 100 nM (presenting higher and stronger binding affinity towards MHC-MHC-II and I) were selected.

#### MHC-I epitopes

From IEDB analysis, 60 MHC-I epitopes were selected based on IC50 values and MHC-I alleles interactions. Finally, five epitopes were obtained, presenting non- allergic behavior and high antigenic score **(**Table 3**).**

**Table 3:** Bepipred linear epitopes (MHC-I) predicted by IEDB server

Number	Start	End	Peptide	Length
1	17	25	RVTQPAAEF	9
2	18	26	VTQPAAEFR	9
3	07	15	KPLPTTITV	9
4	29	37	AHSSLYGRY	9
5	56	64	YLCLRCHQI	9

#### MHC-II epitopes

Total one hundred and five epitopes were selected based of IC50 values. In addition, fifty epitopes were selected among 200 epitopes based on antigenicity, and allergenicity. Finally six epitopes were selected as described above for B cell epitope prediction **(**Table 4**).**

**Table 4:** Bepipred linear epitopes (MHC-II) predicted by IEDB

Number	Start	End	Length	Peptide
1	19	33	15	TQPAAEFRRVAHSSL
2	22	36	15	AAEFRRVAHSSLYGR
3	40	54	15	KCCWFADTNLITCND
4	38	52	15	NCKCCWFADTNLITC
5	37	51	15	YNCKCCWFADTNLIT
6	9	18	9	FRRVAHSSL

### A novel multi epitope vaccine constructions

**Fig. 5:**
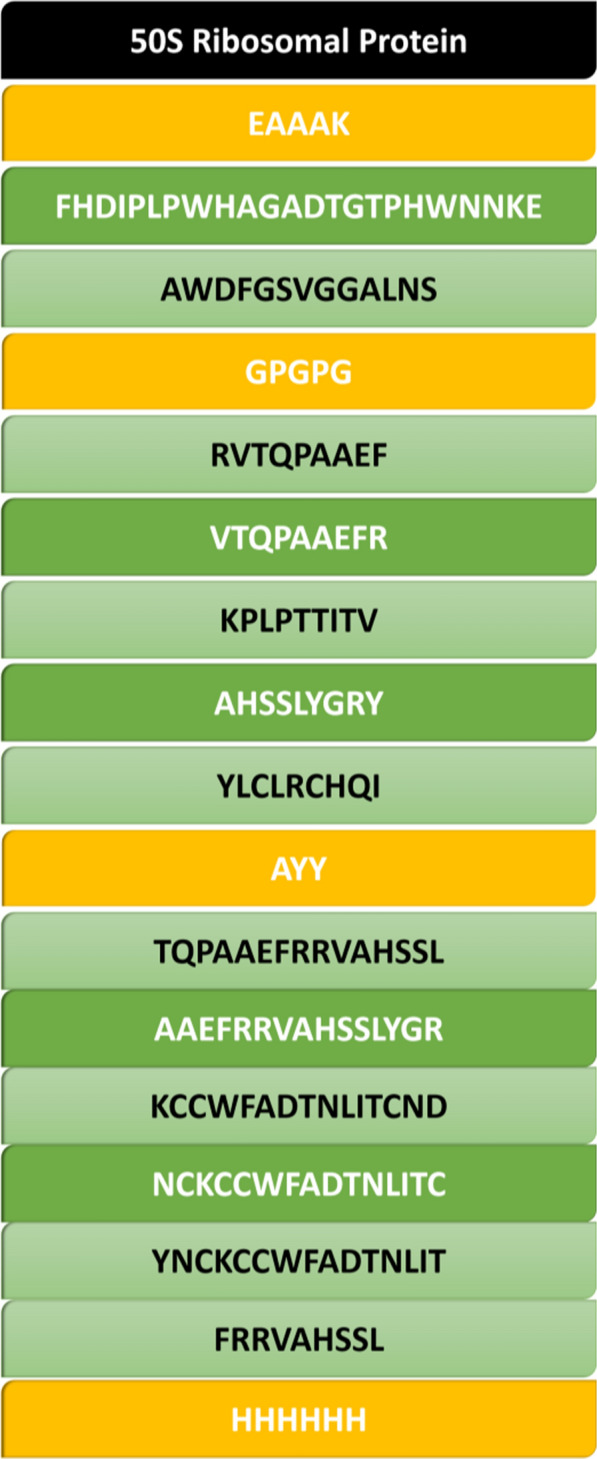
Complete sequence of vaccine construct along with linkers

To construct vaccine, two B-cell epitopes, five MHC-I, and six MHC-II epitopes were used along with linkers (GPGPG, AAY, EAAAK), 50 s ribosomal protein and 6 His tag. At the end of C terminal, the 6 His tag was used, to purify the constructed vaccine **(**Fig. 5**).**

### Physio-chemical characteristics of constructed vaccine

Our results presented the physio-chemical properties of constructed vaccine by IEDB server. Constructed vaccine comprised of amino acids (186), with chemical formula C925H1372N272O256S13_,_ theoretical isoelectric point (8.45), aliphatic index (61.56), molecular weight (20815.52), and instability index (28.00). The instability index value reveals that the constructed vaccine is stable. Whereas the 17 and 13 were presented as number of positively and negatively charged residues respectively.

### Assessment of constructed vaccine

A novel and effective vaccine against *Zika virus* must have good antigenic score, immunogenicity, and should be nonallergic and nontoxic. Therefore, we used different computational approaches to identify the vaccine characteristics. Our results reveal that constructed vaccine has an antigenic score of 0.6552, and is nonallergic predicted by VexiJen 2.0 and AllerTop 2.0 servers. Our data illustrated that the constructed vaccine has good immunogenic and antigenic score and can induce a strong immune response against *Zika virus*.

### Secondary predication of constructed vaccine

**Fig. 6:**
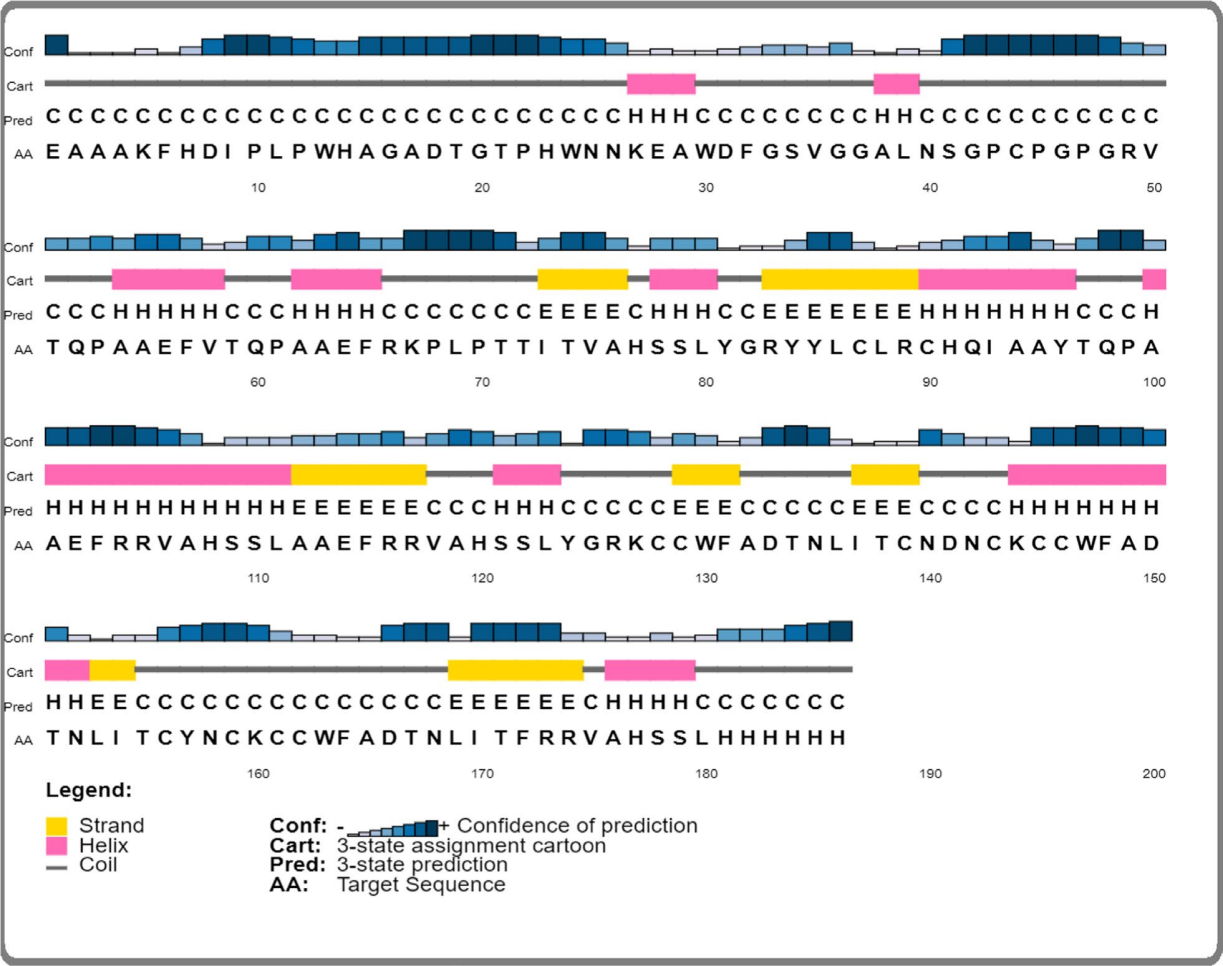
Secondary structure of constructed vaccine. Alpha sheet (46%), beta sheet (33%), and random coil (21%)

The secondary structure of any protein helps to pertain the polypeptide chain, resulting in beta sheet, alpha helix and coil. Our results presented that constructed vaccine comprised of alpha helix (46%), beta-sheet (33%), and coil structure (21%) **(**Fig. 6**).**

#### Three-dimensional structure predication and validation

**Fig. 7:**
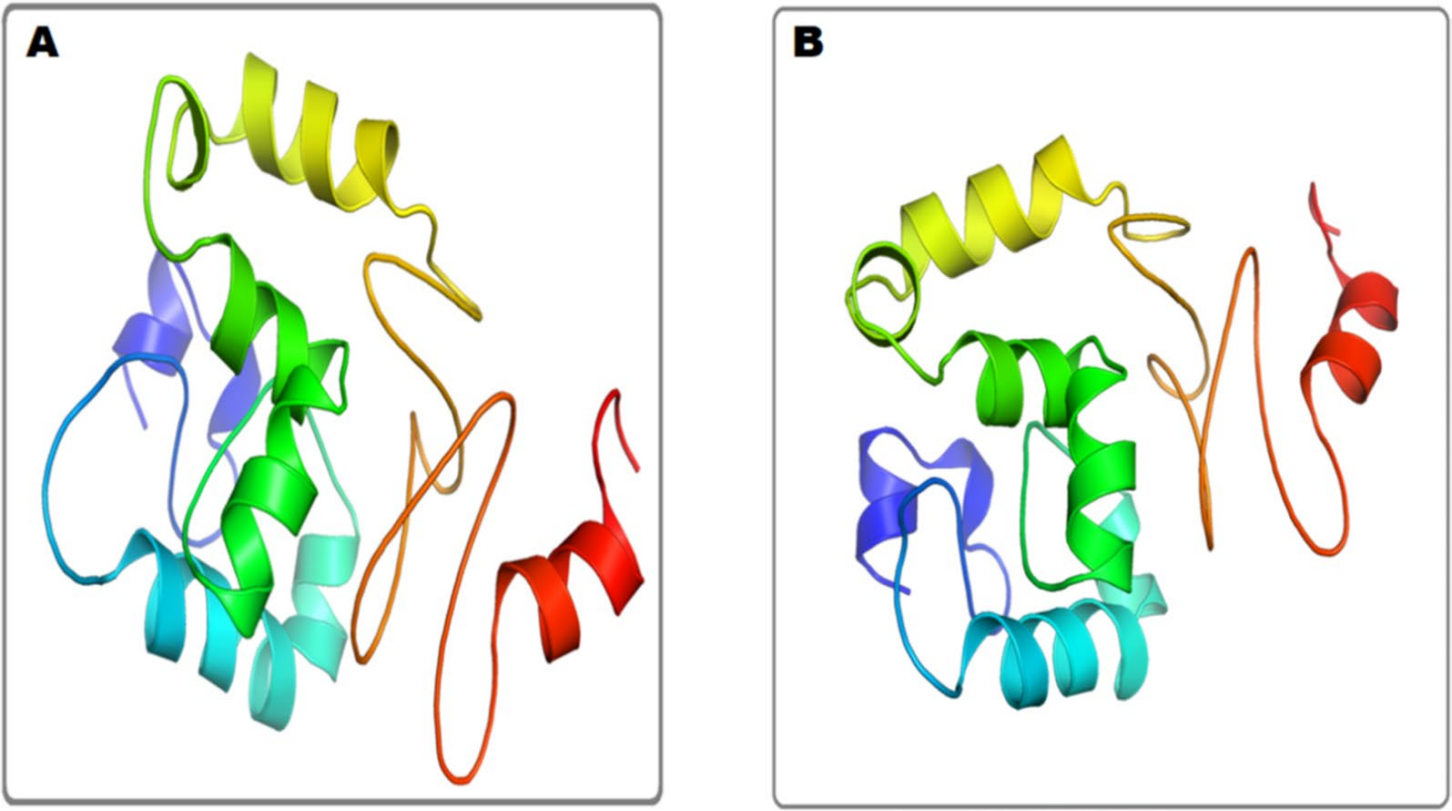
Analysis of three-dimensional structure of vaccine. **A** Predicted 3D vaccine **B**. Refine 3D vaccine

**Fig. 8:**
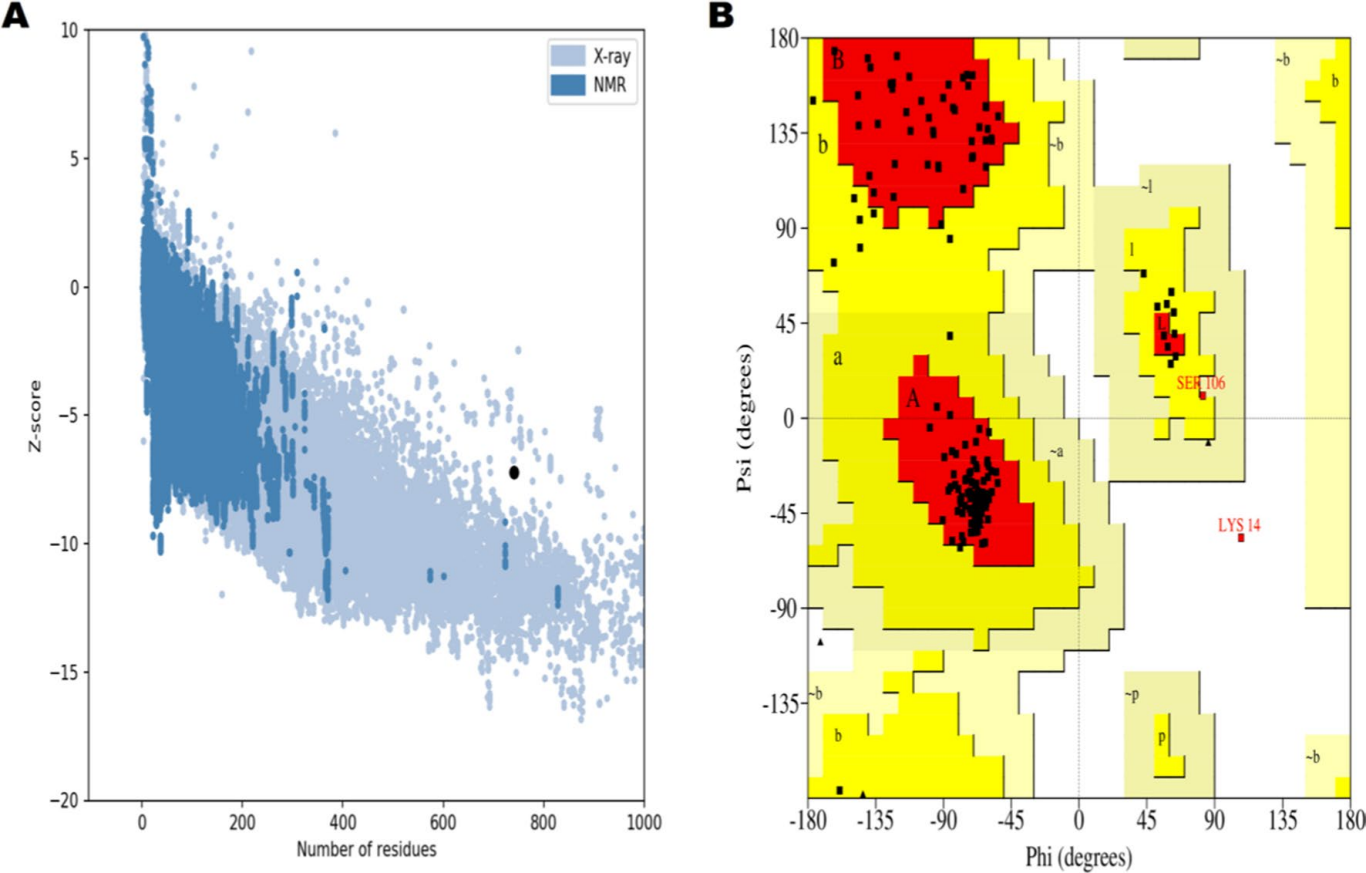
Validation of 3D vaccine structure (**A**). Z-score (-7.89), predicted by ProSA online tool (**B**). Ramachandran Plot for constructed vaccine; favorable region (90.1%), additional region (8.6%) and generously allowed region (0.7%)

Subsequently, the three-dimensional structure of constructed vaccine predicted by trRosetta, as a results, ten models were predicted, in which model 1 was selected on the basis of interaction **(**Fig. 7A**),** and refined by Galaxy Refine **(**Fig. 7B**)**. Furthermore, ProSA-web and PROCHECK server validated the 3D structure. The Z score (-7.89) was predicted by ProSA-web **(**Fig. 8A**).** However, Ramachandran analysis, predicted by PROCHECK server, reveals (0.7%) residues were in generously allowed regions, (90.1%) was in favored and (8.6%) were in additional region **(**Fig. 8B**).**

#### Molecular docking of constructed vaccine with TLR-9

**Fig. 9:**
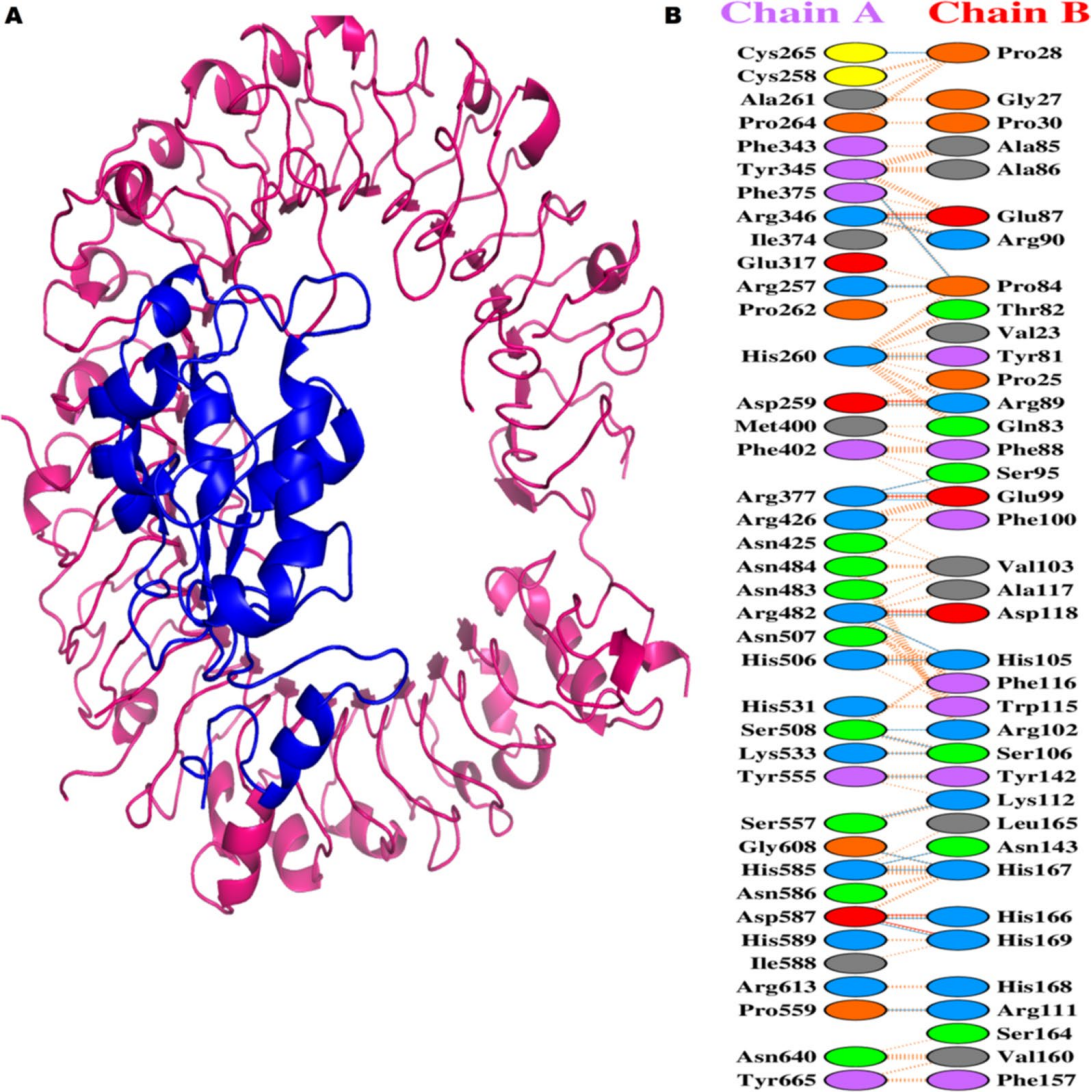
Docking interaction of constructed vaccine (blue) and TLR-9 (megenta) complex by using PDBSum database. (**A**) Constructed vaccine-TLR9 docked complex in cartoon representation. (**B**) All interacting residues of TLR-9 and constructed vaccine. The colours of the residues interacting reflect amino acid properties (Negative: Red, Positive: Blue, Neutral: Green, Aromatic: Pink, Pro&Gly: Orange, Cys: Yellow, and Aliphatic: Grey). Chain A represent the constructed vaccine and Chain B represent TLR-9

Molecular docking was performed with constructed vaccine and TLR-9 by using the ClusPro server. A total ten models were predicted. All ten models were examined by using Pymol program. Interestingly, the model one exhibits good docking score and hydrogen bond interactions. For graphical representation of constructed vaccine/TLR-9 complex, PDBSum online server was utilized **(**Fig. 9**)**. The analysis of constructed vaccine/TLR-9 complex, presented that twenty-four H-bonds, six salt bridges, and two hundred and eighty eight non-bonded contacts were formed. Our results showed the prepared vaccine had excellent performance that triggered strong immune response.

#### Molecular dynamic simulations

**Fig. 10:**
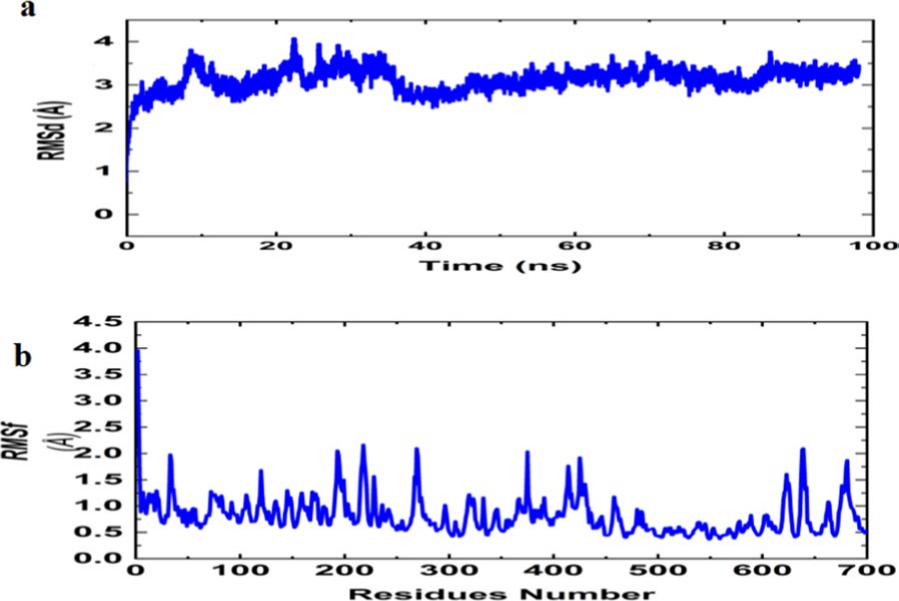
Molecular dynamic simulation of constructed vaccine and TLR-9 complex. (**a**) Root mean square analysis of constructed vaccine and TLR-9 complex. (**b**) Root mean square fluctuation of constructed vaccine and TLR-9 complex

The globe stability of TLR-9/vaccine construct was studied through MD simulation. Root mean square deviations (RMSD) presents structure stability in 100 ns MD simulation period (Fig. 10a). The stability is evaluated by means of Root mean square fluctuation (RMSF) of amino acid residues in TLR-9 receptor. The RMSF results revealed reduced fluctuation, presenting good stability of complex **(**Fig. 10b**).**

#### C-immune simulations

**Fig. 11:**
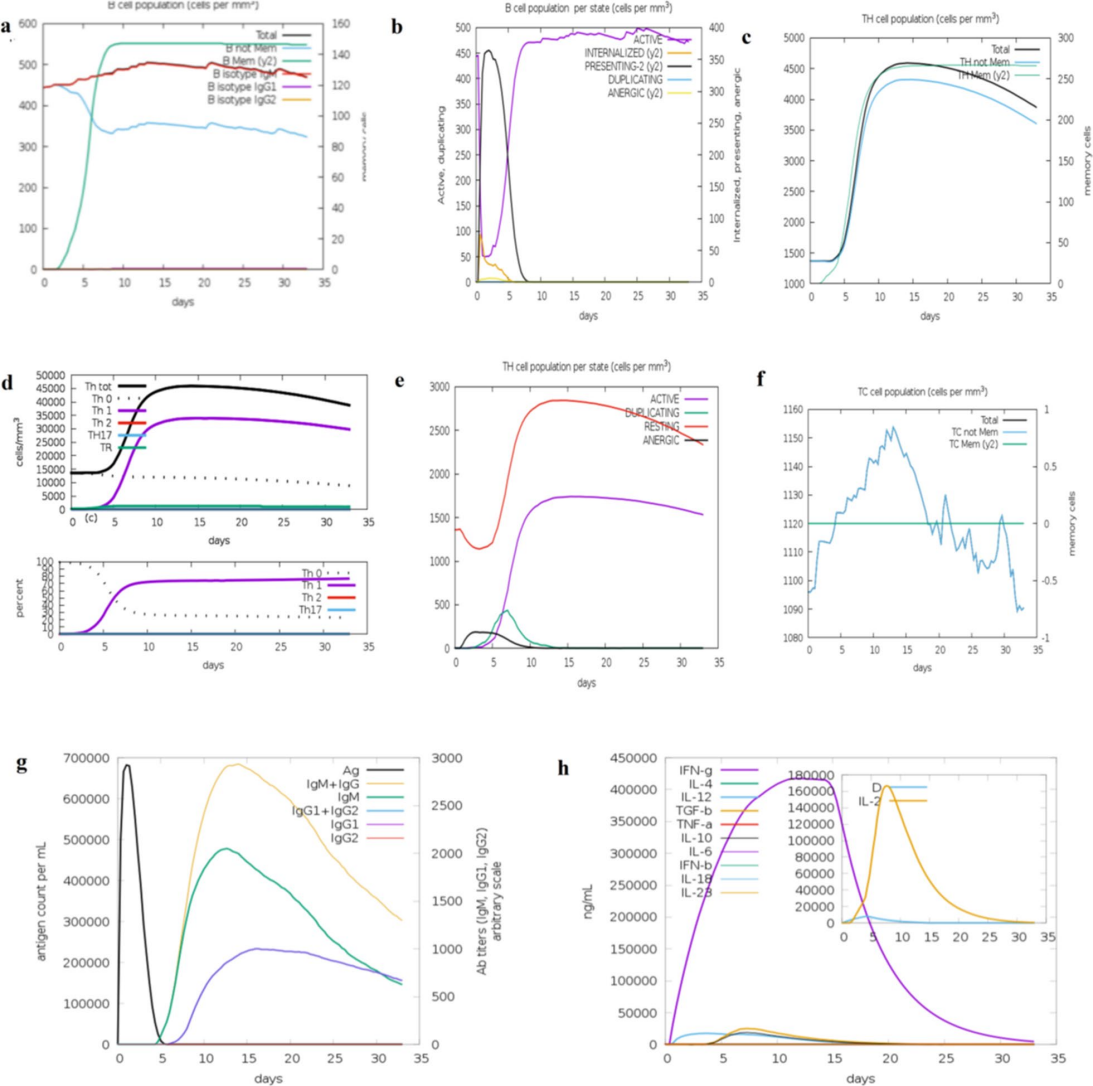
Immune simulations in the host in response to vaccine administration. (**a**) B-cell population. (**b**) Changes in B-cell population and macrophages. (**c**) T helper cell population. (**d**) Significant increase in Th1. (**e**) Population per state of T-helper cell. (**f**) Production of cytotoxic T-cells. (**g**) Antibodies production in response to antigen injection. (**h**) Production of interleukin and cytokines

The C-immune simulation helps to determine the immunological stimulation of vaccine, immune response profile, and immunogenicity. It used machine learning techniques that identify immune responses based on different compartments, including lymph nodes, thymus, and bone marrow Fig. 11). The results present a steady rise in secondary and tertiary response after primary response. Increase in B-cell numbers were found (Fig. 11a, b). Similarly, a marked increase in T-cells were noted (Fig. 11c–f). In addition, Fig. 11g, presented IgM, IgG, IgG1 + IgG2, and IgM + IgG, and elevated amounts of IFN-γ and IL-2 (Fig. 11h).

## Discussion

Currently, the development of computational methods, structure biology, computing tools, constructing methods and biomarker selection are diversified, and plays important roles for the development of an effective vaccine [37]. These advancements have a significant role in understanding the diseases, their mechanisms, and develop effective vaccines for target proteins. There are no such antiviral drugs or commercial vaccines against *Zika virus*. Vaccination is one of the important methods to prevent from infectious diseases [38], including reverse vaccinology, which is used to develop a multi-epitope vaccine, due to its efficiency and cost-effectiveness. Different protective, stable and safe vaccines were designed to prevent infectious diseases [39], including *Burkholderia pseudomallei* [40], *SAR-CoV-2* [41], *Lumpy skin disease* [42], *Monkey Pox* [43], *Helicobacter pylori* [44], *Aeromonas hydrophilla* [45], and *Klebsiella pneumonia* [46]. Therefore, we our study focused to developed a potent multi-epitope based vaccine targeting *Zika virus.* The goal was to develop highly immunogenic vaccine with desirable potential, including nonallergic behavior, nontoxicity, and highly antigenic [47]

Initially, the protein sequence of *Zika virus* was retrieved from NCBI database, and BLAST P was performed. Based on high antigenic score and nonallergic behavior, the protein sequence of *Zika virus* was selected. We evaluated the protein to predict the B-cell epitopes, MHC-I and II epitopes by IEDB server. The epitopes were finalized based on nonallergic behavior, nontoxic behavior and high antigenic score. To construct an efficient multi epitope-based vaccine, different linkers EAAAK, AAY, GPGPG, and adjuvant were joined with B-cell, MHC-MHC-II epitopes and I. These linkers play a crucial role in connecting the prioritized epitopes to construct potent vaccine against *Zika virus*. An ideal vaccine must be antigenic, nontoxic, non-allergenic, and have good immunogenicity. Furthermore, it might induce a robust immune response with no side effects [48]. Our results indicate that the constructed vaccine is an efficient vaccine with good immunogenicity, antigenicity, and non-sensitization. Nontoxicity, and nonallergic. In addition, the secondary structure of the constructed vaccine reveals a fascinating composition, 46%, α-helices, 21% random coils, and 33% extended strands. Naturally, α-helical coil motifs and unfolding area of protein are considered as a type of “structural antigens, and increase in these two structures might helpful to facilitate the antibodies recognition which is induced after infection [49]. Our result presents that the amino acid residues in constructed vaccine were present in preferred area, presenting that quality of model was acceptable. In addition, the three-dimensional structure was constructed by using trRosetta, and its validation was performed by ERRAT, PROSA, and PROCHECK. The physio-chemical characteristics of constructed vaccine were comprised of (186), number of amino acids, chemical formula C_925_H_1372_N_272_O_256_S_13,_ aliphatic index (61.56), and theoretical isoelectric point (8.45). All of our findings and results indicate that the predicted model has great potential for further research.

In the context of immune simulation, various studies suggested an important role of Toll -like receptors. Molecular docking was performed using TLR-9 with the help of ClusPro tool. The molecular docking results reveal strong interaction with TLR9 and a good docking score, indicating its potential to elicit immune response. MD simulation was carried out to understand the stability of constructed vaccine/TLR-9 complex, based on docking interaction. Finally, C-immune simulation was used to predict the stability between constructed vaccine and immunological receptor. The presence of B-cells was observed. The results present a high level of Ig production, IFN-γ, IL-2 and T-cytotoxicity.

A previous study designed a vaccine against Zika virus, in which different vaccines were constructed and predicted antigenic scores were 0.44–0.52 [50]. Interestingly, our constructed vaccine has a score of 0.6552. In addition, Shah et al. generated a chimeric vaccine, model had Z-score − 4.71 predicted by ProSA-web [51], and our vaccine model has a Z-score of -7.89. Christopher et al*.* also generated T-cell epitopes induced by the Zika virus, exhibited significant protection [52]. Moreover, we followed stringent pipeline to evaluate the structural stability, interaction with receptors, and immune responses, using various computational approaches to design an effective vaccine. This work demonstrates that the potent vaccine provided effective protection against *Zika virus.*

## Conclusion

*Zika* virus become major public health problem, there is no effective vaccine and persistent cure for *Zika* infection. Various antiviral drugs have underwent trails however; none of them displayed significant results against this infection. In current the study, we designed multi-epitope-based vaccine, utilizing various immunoinformatics and bio-informatics tools. Our results indicate that the constructed vaccine has good antigenicity, immunogenicity, non-allergy properties, and nontoxicity, and has the ability to trigger the immune responses with no side effects. However, our research enables the validation of this work through comprehensive experimental approach. Therefore, to validate the potency of constructed vaccine, in vivo and in vitro immunological assays are required.

## Data Availability

All the data and its links are available in the manuscript.
